# Genome-Wide Identification of OSC Gene Family and Potential Function in the Synthesis of Ursane- and Oleanane-Type Triterpene in *Momordica charantia*

**DOI:** 10.3390/ijms23010196

**Published:** 2021-12-24

**Authors:** Yutong Han, Ya Yang, Yan Li, Xin Yin, Zhiyu Chen, Danni Yang, Yongping Yang, Yunqiang Yang, Xuefei Yang

**Affiliations:** 1 Key Laboratory of Economic Plants and Biotechnology, Kunming Institute of Botany, Chinese Academy of Sciences, Kunming 650201, China; hanyutong@mail.kib.ac.cn (Y.H.); liyan@mail.kib.ac.cn (Y.L.); chenzhiyu@mail.kib.ac.cn (Z.C.); 2 Key Laboratory for Plant Diversity and Biogeography of East Asia, Kunming Institute of Botany, Chinese Academy of Sciences, Kunming 650204, China; yangya@mail.kib.ac.cn (Y.Y.); yinxin@mail.kib.ac.cn (X.Y.); yangdanni@mail.kib.ac.cn (D.Y.); yangyp@mail.kib.ac.cn (Y.Y.); 3 Plant Germplasm and Genomics Center, Kunming Institute of Botany, Chinese Academy of Sciences, Kunming 650201, China; 4 Institute of Tibetan Plateau Research at Kunming, Kunming Institute of Botany, Chinese Academy of Sciences, Kunming 650201, China; 5 Southeast Asia Biodiversity Research Institute, Chinese Academy of Sciences, Yezin, NayPyiTaw 05282, Myanmar

**Keywords:** triterpenoid, OSC, bitter gourd, biosynthesis

## Abstract

The triterpenes in bitter gourd (*Momordica charantia*) show a variety of medicinal activities. Oxidosqualene cyclase (OSC) plays an indispensable role in the formation of triterpene skeletons during triterpene biosynthesis. In this study, we identified nine genes encoding OSCs from bitter gourd (*McOSC1*–*9*). Analyses of their expression patterns in different tissues suggested that characteristic triterpenoids may be biosynthesized in different tissues and then transported. We constructed a hairy root system in which *McOSC7* overexpression led to an increased accumulation of camaldulenic acid, enoxolone, and quinovic acid. Thus, the overexpression of *McOSC7* increased the active components content in bitter gourd. Our data provide an important foundation for understanding the roles of McOSCs in triterpenoid synthesis.

## 1. Introduction

Triterpenoids are compounds with a carbon skeleton based on six isoprene units. More than 20,000 triterpenoids with nearly 200 different skeletons have been identified in eukaryotic organisms. In plants, triterpenoids represent a category of specialized metabolites with vast structural diversity and pharmacological activities [1].

Since plants contain triterpenoids and other active substances, they have been used as herbal medicines for thousands of years. Triterpenoids in plants function in protection against pests, diseases, and abiotic stresses such as low temperature. Many studies have focused on the diverse pharmacological activities of triterpenoids, such as their anticancer, antiviral, and cholesterol-lowering activities [2]. In addition, due to their unique physicochemical properties, triterpenoids are commonly used in medicinal, agricultural, cosmetics, and food industries as natural emulsifiers, foaming agents, and sweeteners [3,4]. Nonetheless, their low abundance and structural complexity has limited their commercial applications [5,6]. Recent research has shown that triterpenoids can be produced in a heterologous host expressing genes encoding triterpenoid biosynthetic enzymes [7,8,9]. Therefore, analyses of the biosynthetic pathways and metabolic regulation of triterpenoid saponins are vital for understanding and modifying their overall metabolic framework.

The triterpenoid synthesis pathway can be divided into three stages: precursor synthesis, carbon ring skeleton formation, and various complex functional reactions. Isopentenyl diphosphate (IPP), the triterpenoid precursor, is synthesized though two independent pathways: the cytosolic mevalonate (MVA) pathway and the plastidial 2-C-methyl-D-erythritol 4-(dihydrogen phosphate) (MEP) pathway) [10]. Both IPP and dimethylallyl diphosphate (DMAPP) are catalytically converted by geranyl pyrophosphate synthase (GPS) into geranyl pyrophosphate (GPP). Then, IPP and GPP can further form farnesyl pyrophosphate via a reaction catalyzed by methylene pyrophosphate synthase (FPS). Next, under the action of squalene synthase (SQS), two molecules of farnesyl pyrophosphate combine at the head and tail to form squalene, which is then converted into 2,3-oxidosqualene in a reaction catalyzed by squalene cyclooxygenase (SE). In triterpenoid synthesis, the first important step is the cyclization of the common acyclic precursor 2,3-oxidosqualene by oxidosqualene cyclase (OSC) to form various triterpene scaffolds [11]. The chair–chair–chair (C-C-C) conformation produces the dammarenyl intermediate cation, while the chair–boat–chair (C-B-C) conformation forms the protostane intermediate cation, a precursor of sterol, parkeol, and isoarborinol (Figure S1) [12,13,14]. Finally, triterpenoid skeletons are modified by cytochrome P450 monooxygenase (CYP450), UDP-glycosyltransferase (UGT), and glucosidases, forming different types of active triterpenoids. Members of the *OSC* supergene family play crucial roles in the production of triterpene precursors and sterols.

Generally, OSCs can be preliminarily identified by specific structures, specifically, highly conserved sequences such as the DCTAE and QW sequences. The conserved DCTAE sequence is involved in substrate binding and plays an important role in substrate connection and initial cyclization. The QW motif (QXXXXXW), a negatively charged aromatic amino acid region, stabilizes the cationic intermediate during the cyclization of 2,3-oxidosqualene. These repetitive sequences may be related to the structure and function of stable proteins. One of the conserved sequences in OSCs, MWCYCR, is even more highly conserved in β-amyrin synthase (β-AS). The Trp within this domain is highly likely to play an important role in the formation of triterpene skeletons [15]. The MWCYCR sequence not only contains amino acid residues responsible for the determination of product specificity between lupeol and β-amyrin but also a Tyr residue responsible for the formation of pentacyclic triterpenes. The Trp of β-AS regulates the formation of β-amyrin by stabilizing oleanyl cations, while the lack of a Leu residue may terminate the reaction at the lupane-based cationic stage. When the conserved Tyr residue found in all OSCs that produce pentacyclic triterpenes is mutated into a His residue, the reaction products are tetracyclic carbon skeletons instead of pentacyclic triterpenes, inferring the significance of the X site. The X site of this motif harbors His residues, resulting in the generation of tetracyclic products, while Tyr residues result in the generation of pentacyclic products [15].

The roles of certain OSCs in triterpene synthesis have been functionally analyzed in several species, such as *Arabidopsis thaliana*, *Glycine max*, *Gentiana straminea* Maxim, *Panax ginseng,* and *Glycyrrhiza uralensis* [16,17]. Since triterpenes are the main active compounds in bitter gourd (*Momordica charantia* Linn.), recent studies have focused on OSCs in this species. Bitter gourd, also known as bitter melon, kugua, balsam pear, or karela, is an edible and medicinal vine from the Cucurbitaceae family that is cultivated worldwide. It is valued as an edible vegetable and for its multiple medicinal properties [18,19]. The triterpenoids in bitter gourd not only have the potential to prevent and manage diabetes but also have antineoplastic effects. For instance, maslinic acid with high antioxidant activity [20,21] can protect against alcohol-induced liver oxidative stress and inflammatory injury by inhibiting the P38 MAPK pathway [22]. Additionally, it can effectively antagonize lipopolysaccharide/D-galactosamine-induced acute liver injury in mice [23]. Therefore, it is important to identify *OSC* genes from bitter gourd encoding enzymes in the triterpenoid biosynthetic pathway to make better use of them. Takase et al. identified four *OSC*s through RNA-seq analyses and detected their transcript levels in 10 different tissues. Heterologous expression of those four *OSCs* in *Saccharomyces cerevisiae* strain GIL77 revealed their roles in the formation of different substrates for various triterpenes. Diverse triterpene skeletons synthesized by OSCs, including cucurbitadienol, isomultiflorenol, β-amyrin, and cycloartenol, are indicative of the diversity of OSC functions [24]. In the present study, we identified nine *OSC* genes in the genome of bitter gourd, determined their transcript levels in different organs of bitter gourd plants, and explored the role of one of them, *McOSC7*, in triterpene saponin synthesis in bitter gourd using the hairy root system.

## 2. Results

### 2.1. Identification of OSC Gene Family in Bitter Gourd

To identify members of the *OSC* gene family in bitter gourd, BLAST searches were performed at the bitter gourd genome database. The search queries were related protein sequences from multiple species, including *Oryza* *sativa* L., *Panax notoginseng*, *P. ginseng*, and *A. thaliana* (the corresponding NCBI registration numbers are LOC_Os02g04690, LOC_Os02g04710, LOC_Os02g04730, LOC_Os02g04760, LOC_Os06g28820, LOC_Os11g08569, LOC_Os11g18366, LOC_Os11g35710,BAD34645, AJR21209, AJR21210, NP_001292630, AEM42982, ABB76766, NP_001310684, XP_015579761, XP_015581645, XP_015581646, BAA33461, BAA33722, AGG09939, and AIE17466). The sequences of 11 functional *A. thaliana* OSC proteins were downloaded from the TAIR website (https://www.arabidopsis.org/, accessed on 1 June 2020). In this way, highly similar bitter gourd protein sequences were screened as candidate OSCs and were renamed sequentially as McOSC1, McOSC2, McOSC3, McOSC4, McOSC5, McOSC6, McOSC7, McOSC8, McOSC9, McOSC10, McOSC11, and McOSC12. The 12 genes encoding these proteins were further identified based on the annotation results in NCBI. *McOSC11*, *McOSC12*, and *McOSC2* were found to encode the same sequence. *McOSC10* and *McOSC3* were alternative splicing variants. For those sequences identified as alternative splicing variants, the longest one was selected for subsequent analysis. Finally, nine McOSC proteins were identified, comprising 611 (McOSC5) to 768 (McOSC9) amino acids, with theoretical isoelectric points (pIs) ranging from 4.90 (McOSC9) to 4.98 (McOSC5) (Table S2).

To explore the evolution of McOSC proteins, their amino acid sequences were compared with a focus on the two highly conserved sequences: the DCT(A/S)E motif and the QXXXXXW motif (Figure S2). Studies have shown that the highly conserved DCTAE sequence is the substrate binding activity center [25,26]. In McOSC1, McOSC2, McOSC5, and McOSC8, an Ile residue was located two residues upstream from the DCTAE motif, whereas a Val residue was located at that position in McOSC3, McOSC4, McOSC6, and McOSC9. Notably, in contrast with the findings of previous studies, an ILCYCR motif was detected in McOSC5, McOSC6, McOSC7, and McOSC8. The Leu of the MLCYCR/ILCYSR sequence is conserved in lupeol synthases (LUPs). According to annotation information from the NCBI, only McOSC6 is a LUP, and the other three proteins are β-AS-like proteins. The differences in these sequences result in the production of different triterpene metabolites. The alignment analysis revealed three highly conserved repetitive QXXXXXW motifs, which may play an important role in stabilization during catalysis in oxidosqualene cyclases and squalene cyclases across various species. The MWCXCR motif and WVEDPN motif were only present in certain McOSC proteins [24], suggesting that functional differentiation has occurred among this gene family. The analyses also revealed that the amino acid residue at the X site of the MWCXCR motif differs in lanosterol synthase (LAS) (McOSC3), cucurbitadienol synthase (CS) (McOSC1), and cycloartenol synthase (CAS) (McOSC2), while Tyr is present at the X site of β-AS (McOSC4) and isomultiflorenol synthase (IMS) (McOSC9).

### 2.2. Phylogenetic Relationships, Exon–Intron Structure, and Chromosome Location Analysis

**Figure 1:**
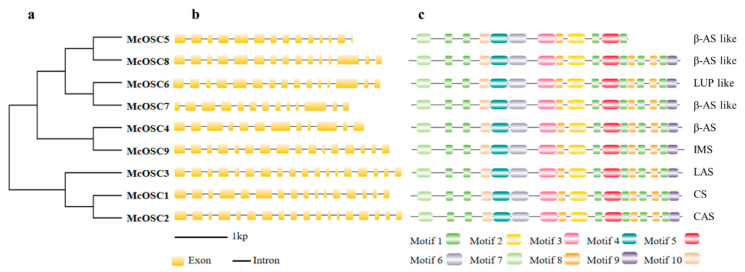
Phylogenetic, exon–intron structure, and motif analysis among nine McOSCs. (**a**) Phylogenetic relationships among nine McOSC proteins; (**b**) exon–intron structure analysis among nine *McOSC* genes; (**c**) motif analysis among nine McOSC proteins. Types of OSCs are β-AS: β-amyrin synthase; LUP: lupeol synthase; IMS: isomultiflorenol synthase; LAS: lanosterol synthase; CS: cucurbitadienol synthase; CAS: cycloartenol synthase.

To explore the phylogenetic relationships of the McOSC family, two neighbor-joining (NJ) trees were constructed: one within McOSC protein sequences (Figure 1a) and the other based on OSC protein sequences from bitter gourd and *A. thaliana* (Figure S3). To analyze the structural characteristics of the McOSCs, we mapped their gene structures, including exons and introns, on the basis of the bitter gourd genome sequence. The results showed that all the McOSCs were intron rich, with 12 to 18 introns (Figure 1b). A conserved motifs analysis of McOSC proteins was conducted using the MEME program to compare the diversity of the motif compositions. Ten conserved motifs were identified (motifs 1 to 10). The structures of proteins encoded by nine *McOSC* genes were similar, with 17 motifs belonging to the 10 types of motifs. Compared with other putative McOSC proteins, McOSC5 lacked five motifs, suggesting that it may have a unique function in the cyclization process, different from that of the others (Figure 1c).

The nine *McOSC*s were mapped to specific chromosomes as a foundation for the further discovery of gene clusters with functional relevance. Three *McOSC*s were located on *M. charantia* chromosome 6 (MC06): *McOSC2*, *McOSC3,* and *McOSC4*. One *McOSC* was mapped to each of MC07 (*McOSC1*) and MC09 (*McOSC9*). The remaining four *McOSC* genes (*McOSC5*, *McOSC6*, *McOSC7*, and *McOSC8*) were mapped to MC08 (Figure S4).

### 2.3. Transcript Profiles of Bitter Gourd OSC Genes in Different Tissues

**Figure 2:**
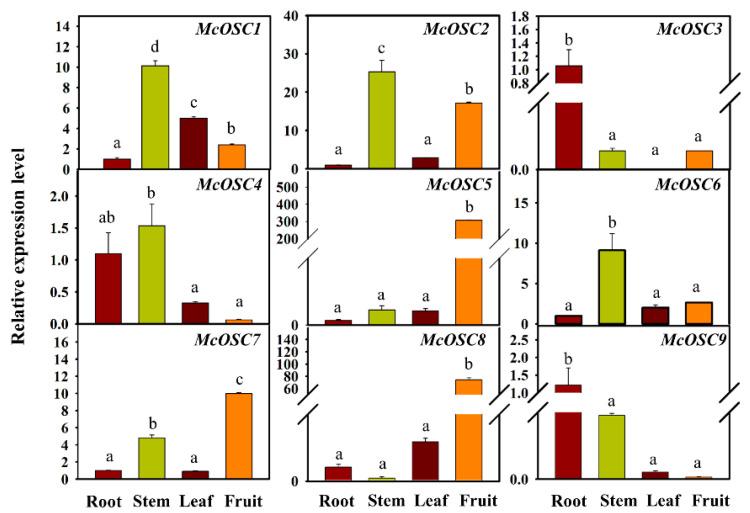
Relative transcript levels of *McOSC*s in different tissues (root, stem, leaf, and fruit) of bitter gourd, as determined by qRT-PCR analyses. Data are means ± SD from three independent experiments. a, b, c, and d indicate significant difference at *p* < 0.05 (independent samples *t*-test). Statistical analysis was performed using IBM SPSS Statistics 20.0.

To better understand the functional divergence of McOSCs, the transcript levels of their encoding genes in different tissues of bitter gourd were assessed. According to the results of qRT-PCR analyses of root, stem, leaf, and fruit samples, the highest transcript levels of *McOSC1*, *McOSC2*, and *McOSC6* were in the stem, and their transcript levels were lower in other analyzed tissues, with the lowest levels in the root. The transcript level of *McOSC4* was highest in the stem and lowest in the fruit. The highest transcript levels of *McOSC3* and *McOSC9* were in the root, while those of *McOSC5*, *McOSC7*, and *McOSC8* were in the fruit (Figure 2). Overall, members of the *McOSC* gene family showed high tissue specificity in their transcriptional patterns, indicating that the triterpenes synthesized by their encoded McOSCs may also show different distributions among tissues.

### 2.4. Widely Targeted Metabolomics Analysis of Transformed Hairy Roots of Bitter Gourd

**Figure 3:**
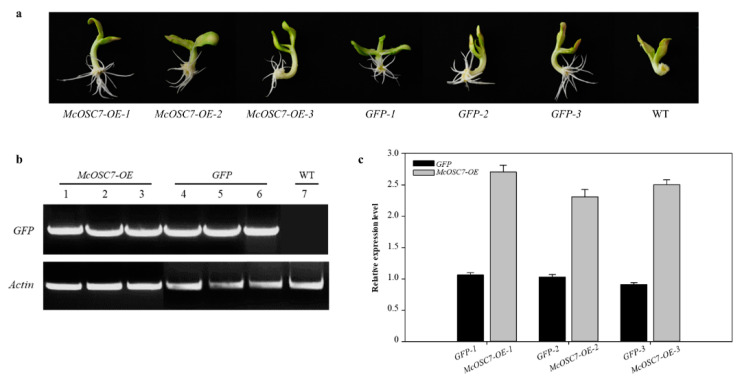
Identification of bitter gourd hairy roots and gene transcript level analysis. (**a**) Pictures of *McOSC7* overexpression (*McOSC7*-*OE*), *GFP* empty, and uninfected bitter gourd (WT) roots; (**b**) Results of PCR analyses of *Actin* in *McOSC7*-*OE*, *GFP* empty, and WT roots; (**c**) Relative gene transcript levels in *GFP* and *McOSC7*-*OE* transgenic materials.

To establish a bitter melon system that accumulates functional triterpenoids, cotyledon explants were transformed with *Agrobacterium* harboring the *McOSC7* sequence. The positive hairy root transformants were verified by PCR amplification of the heterologous sequence. Explants that were not infected did not grow roots in subsequent cultures, so bitter gourd roots with empty vector (*GFP*) were used as the blank control (Figure 3a,b). The results of hairy root qRT-PCR analysis confirmed that *McOSC7* was overexpressed in transformed hairy root material, with an average 2.5-fold increase in its transcript level over that in the blank control (Figure 3c).

**Figure 4:**
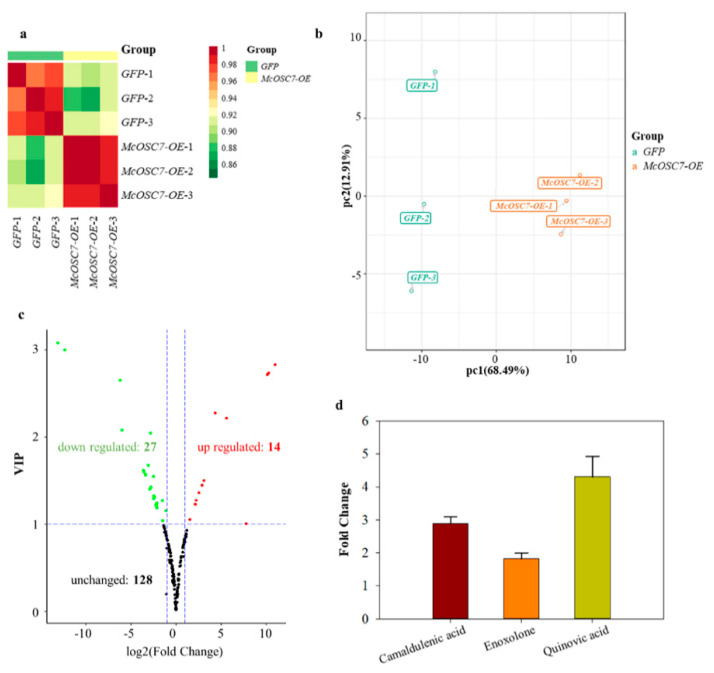
Widetarget metabolic data assessment and differentially accumulated metabolites (DAMs) analysis. (**a**) Pearson’s correlation coefficients among the *McOSC7*-*OE* samples and quality control sample (*GFP*); (**b**) The quality control PCA score chart. The same color represents three biological replicates of the same sample; the x-axis represents the first principal component and the y-axis represents the second principal component; (**c**) Volcano plot showing the differential metabolites in *GFP* vs. *McOSC7*-*OE*, VIP means variable importance in project; (**d**) Fold change of camaldulenic acid, enoxolone, and quinovic acid in *McOSC7*-*OE* transgenic hairy roots, data represent the mean ± SD of three independent experiments.

After confirming this significant increase in *McOSC7* expression, we conducted a widely targeted metabolomics analysis to detect dynamic changes in metabolites in the transgenic hairy roots. All treatments were performed with three independent cultures (biological repetitions), with hairy roots harboring the empty *GFP* vector as the control. Based on comparisons of mass spectrometry data with those in the local metabolic database, the metabolites of the samples were analyzed qualitatively and quantitatively. Figures S5 and S6 show the total ions current (TIC) diagram of mixed QC samples (i.e., the sum of the intensity of all ions in the mass spectrum at each time point) and the multi-peak diagram of MRM metabolite detection (XIC of multi-substance extraction), respectively. In these figures, different metabolites are represented by differently colored peaks. The repeatability among intragroup samples was verified by Pearson’s correlation analyses (Figure 4a). The results of principal component analysis (PCA) showed a trend of metabolome separation between groups, indicating that the metabolomes differed between the hairy roots overexpressing *McOSC7* (*McOSC7-OE*) and those expressing only *GFP* (Figure 4b). We identified 169 metabolites, including ten triterpenes, in bitter gourd hairy roots (Table S3).

Significantly differentially accumulated metabolites (DAMs) were screened according to the following criteria: fold change ≥1.5 and ≤0.5, and a variable importance projection value (VIP) of ≥1 in the orthogonal partial least squares discriminant analysis (OPLS-DA) model. Under these screening conditions, 41 DAMs were detected in *McOSC7*-*OE* materials, including 27 downregulated and 14 upregulated DAMs (Figure 4c). Among them, three triterpenoids were markedly upregulated in the hairy root materials: cucurbitacin D and enoxolone, which were present at levels 1.43 and 1.82 times higher, respectively, in the *McOSC7-OE* materials than in *GFP*; and quinovic acid, which was present at a level 4.31 times higher in *McOSC7-OE* than in *GFP* (Figure 4d, Table S3).

## 3. Discussion

In the biosynthesis of triterpenoids in plants, the cyclization of 2,3-oxidosqualene catalyzed by OSC represents the first committed step of triterpene diversification [27], generating more than 100 different triterpene skeletons [28]. To date, more than 80 OSCs have been identified and functionally characterized in plants, mainly via the heterologous expression of cDNAs in appropriate yeast strains. In this study, we identified nine *McOSC* genes in bitter gourd, including four *OSC*s whose functions have been reported previously [24], and five new ones (*McOSC3*, *McOSC5*, *McOSC6*, *McOSC7*, and *McOSC8*) (Table S2). The results of sequence alignment analyses suggested that *McOSC3* and *McOSC6* encode LAS and LUP, respectively, while *McOSC5*, *McOSC7*, and *McOSC8* encode β-AS-like enzymes with similar catalytic functions.

Gene structure, to some extent, is closely related to the function of the encoded product. In the phylogenetic tree analysis, *McOSC*s were mainly divided into two branches (Figure 1): one branch contained genes encoding β-AS, LUP, and IMS-type enzymes generating the most common pentacyclic triterpenoid skeletons via a C-C-C conformation dammarenyl cation path and the other contained genes encoding LAS, CAS, and CS-type enzymes forming tetracyclic skeletons via a C-B-C conformation prosteryl cation path. This explains the significant similarity between the structure of cucurbitacins and steroidogenic triterpenes. Cucurbitacins, which are widely distributed in Cucurbitaceae plants, have a variety of pharmacological activities such as anticancer and anti-inflammatory activity [29]. Therefore, it is of great value to explore the mechanisms of cucurbitadienol formation. One strategy to increase the accumulation of active cucurbitacins in bitter gourd is to functionally modify OSCs by altering the amino acid residues involved in triterpene product specificity [30]. The catalytic activity of OSCs is mainly controlled by a few key amino acids located at its active center. Therefore, to improve the characteristics of the enzyme, it is important to understand the relationships among the position of amino acids, the structure and function of OSCs, and their catalytic mechanism.

Several studies have shown that *OSC* family genes show distinct expression patterns in different tissues of bitter gourd and that their encoded products play diverse biological roles in resistance to pests and diseases and in adaptation to the ecological environment [31]. A previous study found that the expression level of *McCBS* was highest in leaves and lowest in fruits [32], which was not expected. Thus, the gene transcript level may not correspond to the final levels of the reaction product in some tissues because of transport of triterpenoids among tissues. In the present study, we detected higher transcript levels of *McOSC1* (encoding CS) in stems than in leaves. This may have been because we collected fruit at a different stage. If the transcript levels of *McOSC*s change dynamically during development, it should be possible to select an appropriate collection time to maximize the triterpenoid content and control the degree of bitterness.

In this study, the overexpression of *McOSC7* in a bitter gourd hairy root system markedly increased the contents of camaldulenic acid, enoxolone, and quinovic acid. Among the three pentacyclic triterpenoids with rich pharmacological activities, enoxolone has been used to treat chronic hepatitis patients in China and Japan for more than 20 years [33], on account of its protective effect against livery injury. Pharmacological studies have proved that enoxolone has anti-arthritis, anti-allergy, anti-virus, anti-estrogen, anti-leukocyte formation, and anti-cancer properties [34], and it has shown satisfactory therapeutic effects in the treatment of many diseases. It follows that the overexpression of *McOSC7* greatly improved the medicinal value of bitter gourd through increasing the levels of three important triterpenoids. We detected ursane- and oleanane-type triterpenes as end products of McOSC7, suggesting that it is a multifunctional AS. Interestingly, a previous study also found that most of the identified OSCs capable of producing α-amyrin were multifunctional, generating more than one product, often including β-amyrin [35].

## 4. Materials and Methods

### 4.1. Identification of OSC Gene Family in Bitter Gourd

To identify members of the *OSC* gene family in bitter gourd, we performed BLAST searches at the bitter gourd database (https://www.ncbi.nlm.nih.gov/genome/?term=momordica+charantia, accessed on 1 May 2020). The search queries were the sequences of previously identified OSCs in *P. ginseng*, *A. thaliana*, *P. quinquefolius*, *O. sativa*, and other species [36]. Genes with high similarity were identified as candidate *McOSC*s. The ProtParam tool of ExPaSy (https://web.expasy.org/protparam/, accessed on 1 May 2020) was used to calculate physicochemical parameters including the grand average hydropathicity (GRAVY), theoretical isoelectric point (pI), number of amino acids, and relative molecular weight (MW).

### 4.2. Phylogenetic Relationships, Exon–Intron Structure Analysis, and Chromosome Location

The OSC protein sequences were aligned using MAFFT version 7.0, and a codon matrix was generated using the MUSCLE alignment package in MEGA7.0 [37]. The evolutionary history was inferred using the neighbor-joining method. The bootstrap consensus tree inferred from 1000 replicates was taken to represent the evolutionary history of the analyzed taxa. The intron/exon structures of *OSC* genes were analyzed using the online Gene Structure Display Server (http://gsds.cbi.pku.edu.cn/, accessed on 1 June 2020) [38]. Thereafter, the MEME program was used to search for conserved motifs in the McOSC protein sequences (http://meme-suite.org/tools/meme, accessed on 1 June 2020) [39]. Mapinspect was used to visually analyze the chromosomal location of *OSC*s based on the bitter gourd genome [40].

### 4.3. Plant Materials

Seeds of bitter gourd were sown in nutrient soil and germinated in the dark in a greenhouse at 23 °C. After germination, the plants were moved to an artificial climate room (25 °C days/8 °C nights; 16 h light/8 h dark photoperiod with light supplied at 200 mmol photon m^−2^s^−1^, relative humidity 70–80%). After 3–4 weeks of growth, the plants were harvested, separated into roots, stems, and leaves, and then, each part was washed and dried. Then, all samples were immediately frozen in liquid nitrogen and stored at −80 °C until analysis. Different tissues were sampled with three replicates for qRT-PCR analyses.

The seeds were sterilized by immersion in 5% (*v*/*v*) NaClO (Tianjin Fengchuan Chemical Reagent Co., Ltd., Tianjin, China) with a drop of Tween 20 (NeoFroxx GmbH, Einhausen, Germany for 5 min and then rinsed in sterile distilled water until no foam appeared and the water was clear. Five seeds were aseptically placed on MS basal medium (per L: 4 g/L M519 powder (PhytoTechnology Laboratories & trade, Lenexa, Kansas, USA), 30 g sucrose (Sangon Biotech, Shanghai, China), 7 g agar (Sangon Biotech, Shanghai, China), pH 5.8, sterilized by high-temperature sterilization). The seeds were incubated in the dark until germination, and then, the seedlings cultured under light until the cotyledons fully developed. The cotyledons were used as explants. The explants were cultured on callus-induction medium (MS medium containing 0.75 mg/mL 6-benzyl amino purine and 0.2 mg/mL naphthalene acetic acid) for 4 days to obtain materials for the genetic transformation of hairy roots.

### 4.4. RNA Extraction and qRT-PCR

Total RNA was isolated using the Eastep^®^ Super Total RNA Extraction Kit (Promega, Madison, WI, USA). The extracted RNA was quantified with a NanoDrop1000 spectrophotometer (NanoDrop Technologies, Inc., Wilmington, DE, USA), and its integrity was checked by 1% agarose gel electrophoresis. Approximately 5 μg RNA was reverse-transcribed using the GoScript Reverse Transcription System (Promega, Madison, WI, USA) to generate cDNA. All of the primers were designed using Primer-BLAST (https://www.ncbi.nlm.nih.gov/tools/primer-blast/index.cgi?LINK_LOC=BlastHome, accessed on 1 June 2020) with the following parameters: 150–200 bp polymerase chain reaction (PCR) product size, Nr database, 55–61°C primer Tm, and bitter gourd as the ‘Organism’. The bitter gourd *β-tubulin* was amplified as an internal control. All primers used for qRT-PCR are listed in Table S1. Using FastStart Universal SYBR Green Master Mix (Rox, Roche, Indianapolis, IN, USA) and a 7500 Sequence Detection System (Applied Biosystems, Foster, CA, USA), qRT-PCR was conducted in triplicate with different cDNAs synthesized from three biological replicates of different tissues. The reaction parameters for thermal cycling were as follows: 95 °C for 10 min, followed by 40 cycles of 94 °C for 5 s and 60 °C for 15 s. To analyze the transcript abundance of each reference gene, we examined the cycle threshold (CT) values of candidate reference genes, as the relative abundance of these genes can be reflected by the cycle threshold. The expression value for each gene was calculated using the 2^−ΔΔc_t_^ method [41]. The relative gene transcript levels were obtained by dividing the extrapolated transcript levels of the target genes by the transcript level of the internal control from the same sample. The significance of the difference between the treatment and the control was determined by independent-samples *T* test.

### 4.5. Agrobacterium-Mediated Transgenic Hairy Roots of Bitter Gourd

*McOSC7* was cloned and introduced into the *pRI101-GFP* vector with a One-Step Cloning Kit (Vazyme, Nanjing, China) according to the manufacturer’s instructions. After confirmation by DNA sequencing, the recombinant plasmid (*pRI101-GFP*-*McOSC7*) and the empty plasmid (*pRI101-GFP*, the blank control) were separately transformed into *Agrobacterium rhizogenes* LBA9402. After being infected with LBA9402, the cultured sterile cotyledons were cultured on MS solid medium for 2 days. Then, the cotyledons were rinsed with 1/2 MS culture solution containing 1 mg/mL cephalosporin and transferred to MS solid medium containing 500 mg·L^−1^ TMT. Hairy roots began to grow after approximately 10 days [42]. The induced hairy roots were observed and photographed under a laser confocal microscope to document heterologous gene expression. Thereafter, the roots were washed to remove agar and then ground in liquid nitrogen before extracting DNA using the CTAB method. The negative control consisted of DNA extracted from uninfected sterile seedling roots. Positive transgenic hairy roots were identified by PCR amplification using the following pair of specific primers designed based on the selectable GFP marker: GFP-F (5′→3′): GACAGTGGTCCCAAAGATGG; GFP-R (5′→3′): CCTCCTTGAAGTCGATGCCC). The PCR products were sent to Shanghai Biotechnology Sequencing Co., Ltd. (Shanghai, China) to ensure the successful transfer of *McOSC7* before subsequent analyses.

### 4.6. Widely Targeted Metabolic Profiling and Statistical Analysis

Metabolite profiling was carried out using a widely targeted metabolome method by Wuhan Metware Biotechnology Co., Ltd. (Wuhan, China) (http://www.metware.cn/, accessed on 1 July 2020) [43]. Freeze-dried roots were crushed at 30 Hz for 1.5 min using a mixed mill with zirconia beads (MM 400, Retsch, Haan, Germany). A 100 mg portion of the obtained powder was extracted with 1.0 mL aqueous methanol (70:30 methanol/water) at 4 °C overnight. After centrifugation at 10,000 g for 10 min, the extract was passed through a CNWBOND Carbon-GCB SPE column (250 mg, 3 mL; ANPEL, Shanghai, China) and filtered before HPLC-MS analysis (SCAA-104, 0.22 pore size (μm); ANPEL, Shanghai, China). The sample extracts were analyzed using an LC-electrospray ionization-MS/MS system. Details of the analytical conditions for HPLC and ESI-Q TRAP-MS/MS have been provided elsewhere [44].

Software Analyst 1.6.3 was used to process mass spectrum data. Based on MWDB (Metware Database) and public metabolite information databases, the primary and secondary mass spectra data were analyzed qualitatively. In qualitative analyses of some substances, the isotope signals, some repeated signals (containing K^+^, Na^+^, NH4^+^), and fragments of other substances with larger molecular weight were removed. The structure analysis of metabolites was conducted by consulting public mass spectrum databases, such as MassBank (http://www.massbank.jp/, accessed on 1 July 2020), KNAPSAcK (http://kanaya.naist.jp/KNApSAcK/, accessed on 1 July 2020), HMDB (http://www.hmdb.ca/, accessed on 1 July 2020), MoTo DB (http://www.ab.wur.nl/moto/), and METLIN (http://metlin.scripps.edu/index.php, accessed on 1 July 2020). The metabolites were quantified by multiple reaction monitoring (MRM) using triple quadrupole mass spectrometry [45]. The characteristic ion of each substance was screened by triple quadrupole LC-MS/MS, and the signal intensity (CPS) of the characteristic ion was measured by the detector. MultiQuant software was used to manage the mass spectrometry files and to integrate and correct chromatographic peaks. The peak area (Area) of each chromatographic peak represented the relative content of the corresponding substance (as a proportion of the total of all peak areas in each sample).

Before analyses of significant differences in metabolite accumulation among samples, a PCA was performed for all samples (including quality control samples) to visualize the overall metabolic differences between the groups and within each group. Orthogonal partial least squares discriminant analysis (OPLS-DA) was used to decompose the X matrix information into two types of information related to Y and irrelevant information. The difference variables were screened by removing irrelevant differences. Differentially accumulated metabolites were screened using the following criteria: variable importance in project (VIP) ≥ 1 and fold change ≥2 or ≤0.5.

## 5. Conclusions

**Figure 5:**
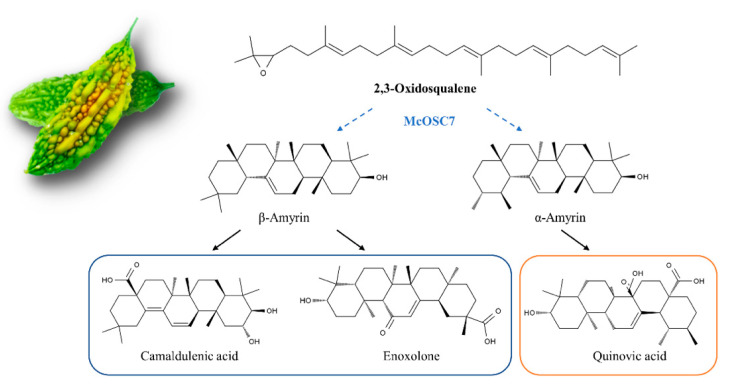
Effect of bitter gourd *McOSC7* on triterpene synthesis. Overexpression of *McOSC7* in hairy roots increased the contents of camaldulenic acid, enoxolone, and quinovic acid. Dotted arrows represent possible intermediates; solid arrows represent detected final products.

In this study, nine *McOSCs* were identified from bitter gourd, and their gene structures were analyzed. These results provide the basis for further research on amino acid substitutions that can significantly alter OSC products. The nine genes were expressed at different levels in different tissues, suggesting that different characteristic triterpenoids might be biosynthesized in different tissues. *McOSC7* was successfully overexpressed in a bitter gourd hairy system, where it promoted the accumulation of camaldulenic acid, enoxolone, and quinovic acid (Figure 5). Thus, our results demonstrate that the overexpression of an *OSC* gene can improve the pharmacological value of bitter gourd. Further research is required to explore the specific synthesis mechanisms of ursane- and oleanane-type triterpenoids in bitter gourd.

## Supplementary Materials

The following are available online at https://www.mdpi.com/article/10.3390/ijms23010196/s1.

## Abbreviations

OSC	oxidosqualene cyclase
qRT-PCR	quantitative real-time PCR
cDNA	coding DNA
Tm	melting temperatures
TMT	timentin
HPLC-MS	high-performance liquid chromatography—mass spectrometry
MS	murashige and skoog
ESI	electrospray ionization
Q TRAP	triple quadrupole linear ion trap mass spectrometer
