# Supplementary material for: Genome-Wide Identification of OSC Gene Family and Potential Function in the Synthesis of Ursane- and Oleanane-Type Triterpene in Momordica charantia
Source: Int J Mol Sci. 2021 Dec 24;23(1):196. doi: 10.3390/ijms23010196 (PMC8745088; doi:10.3390/ijms23010196)
Supplement: Supplementary file 1 [file ijms-23-00196-s001.zip › ijms-1492048-supplementary.pdf]

Genome-wide identification of OSC gene family and potential function in the synthesis of ursane- and oleanane-type triter-pene in *Momordica charantia*

Yutong Han [a,b,c,d,f,1], Ya Yang[b,c,d,1], Yan Li [a,b,c,d,f,1], Xin Yin [b,c,d,f], Zhiyu Chen [a,b,c,d,f], Danni Yang [b,c,d,f], Yongping Yang [b,c,d], Yunqiang Yang [b,c,d]*, Xuefei Yang [a,b,e]*

[a] Key Laboratory of Economic Plants and Biotechnology, Kunming Institute of Botany, Chinese Academy of Sciences, Kunming 650201, China

[b] Key Laboratory for Plant Diversity and Biogeography of East Asia, Kunming Institute of Botany, Chinese Academy of Science, Kunming, 650204, China

[c] Plant Germplasm and Genomics Center, Kunming Institute of Botany, Chinese Academy of Sciences, Kunming 650201, China

[d] Institute of Tibetan Plateau Research at Kunming, Kunming Institute of Botany, Chinese Academy of Sciences, Kunming 650201, China

[e] Southeast Asia Biodiversity Research Institute, Chinese Academy of Sciences, Yezin, NayPyiTaw 05282, Myanmar

[f] University of Chinese Academy of Sciences, Beijing 100049, China

[1] These authors contributed equally to this work.

* Correspondence: Yunqiang Yang, yangyunqiang@mail.kib.ac.cn; Tel.: 86-871-65230873. Xuefei Yang, xuefei@mail.kib.ac.cn; Tel.: 86-871-65223398

a

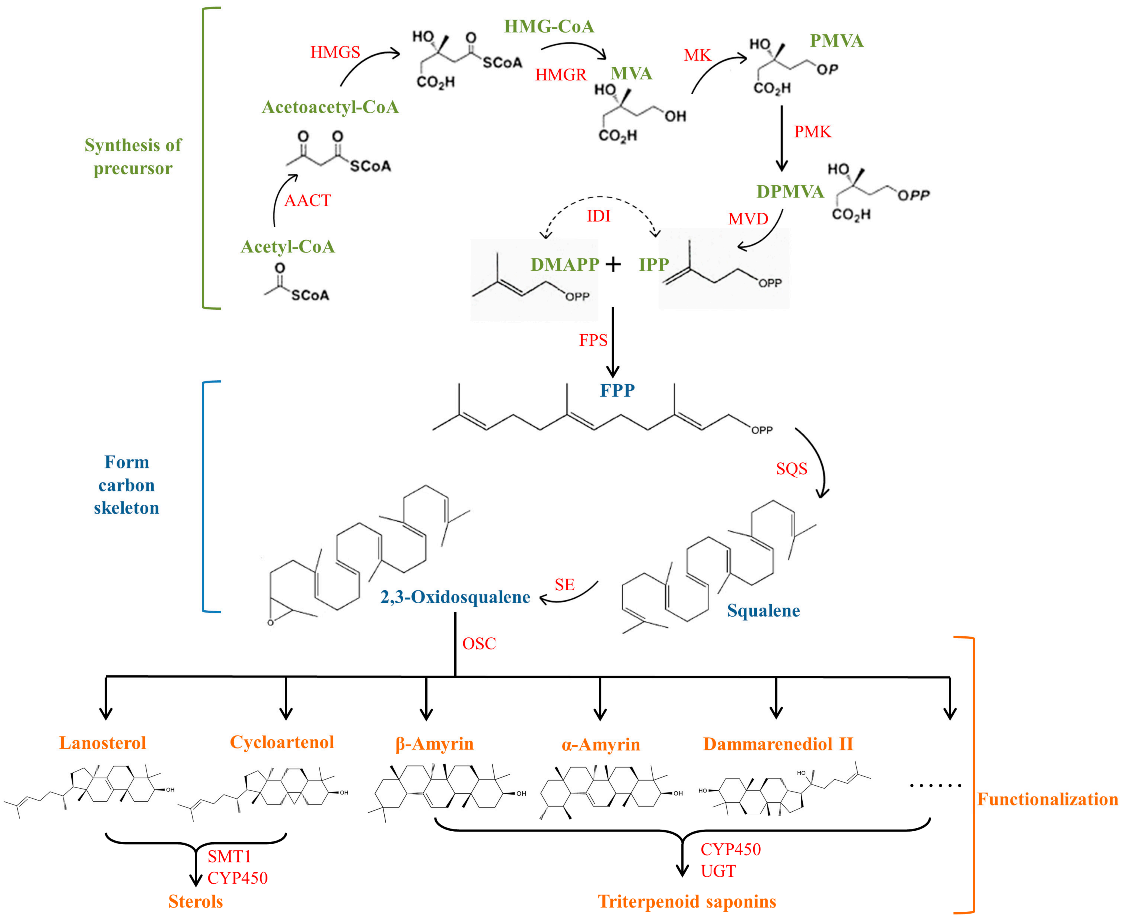

b

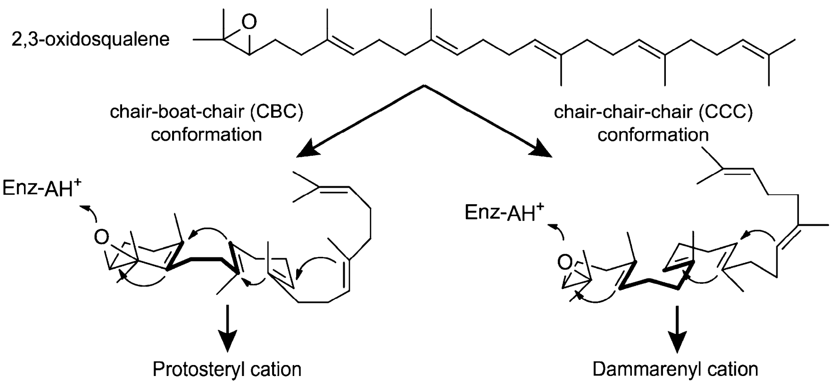

Figure S1 Synthesis pathway of triterpenoids. (**a**) Triterpenoids are synthesized by the isoprenoid pathway, which can be generally divided into three stages: precursor synthesis, carbon ring skeleton formation and various complex functional reactions; (**b)** Conformational arrangement of 2,3-oxidosqualene [1].

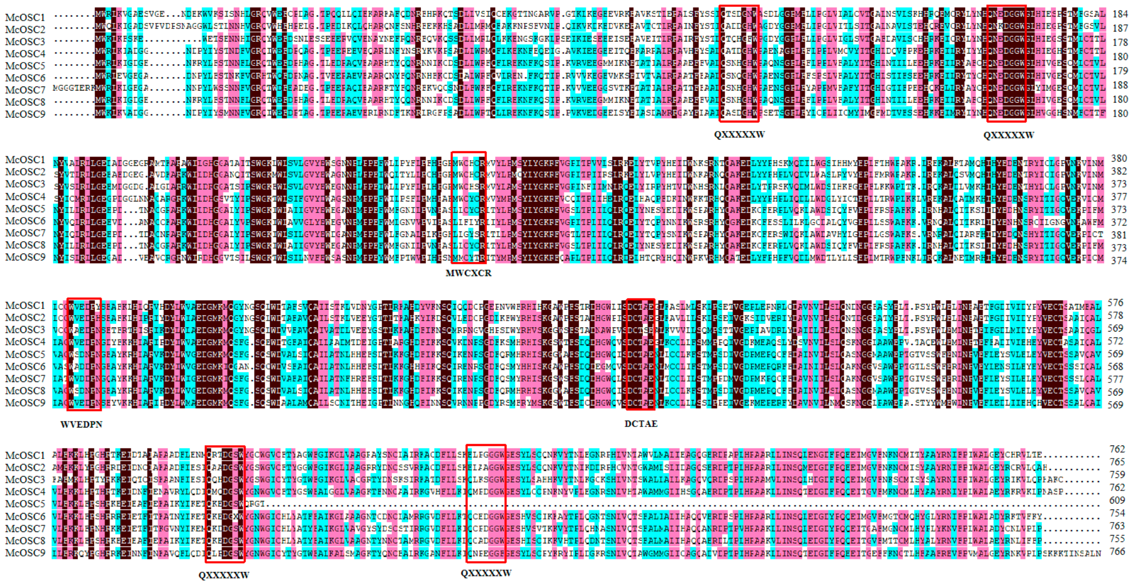

Figure S2 Conserved domain identification of nine McOSC genes. The red boxes in the figure are the conserved sequences of the OSCs gene family DCTAE, QXXXXXW, WVEDPN and MWCYCR.

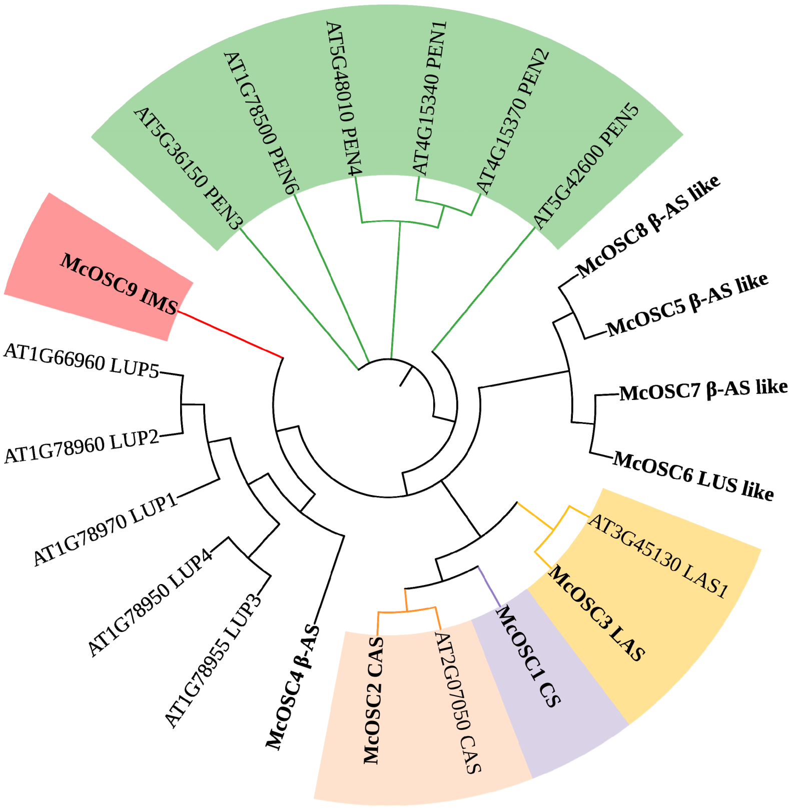

Figure S3 Phylogenetic analysis of nine McOSC genes with AtOSC genes from *Arabidopsis thaliana*. Genes in bold is OSCs from bitter gourd. β-AS: β-Amyrin synthase; LUP: Lupeol

synthase; CAS: Cycloartenol synthase; CS: Cucurbitadienol synthase; IMS: isomultiflorenol synthase; PEN: pentacyclic triterpene synthase.

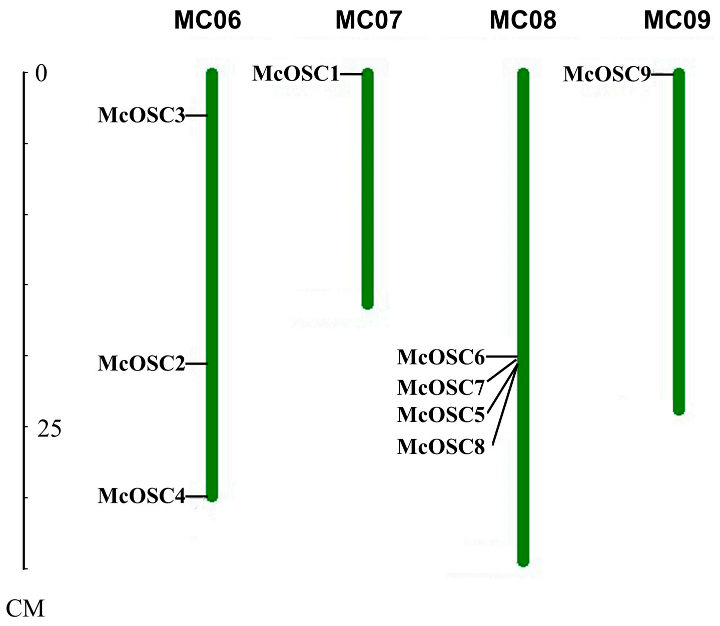

Figure S4 Chromosome location analysis of nine McOSC genes in bitter gourd. The chromosome is shown in green, and the McOSC genes are marked on a line at the approximate position.

a

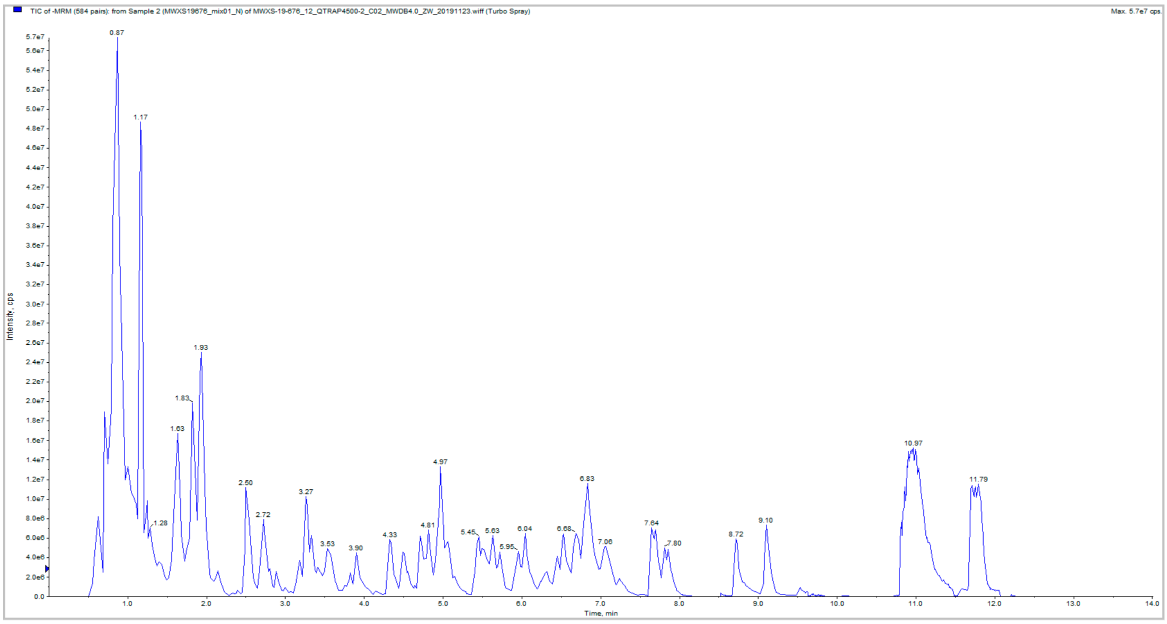

b

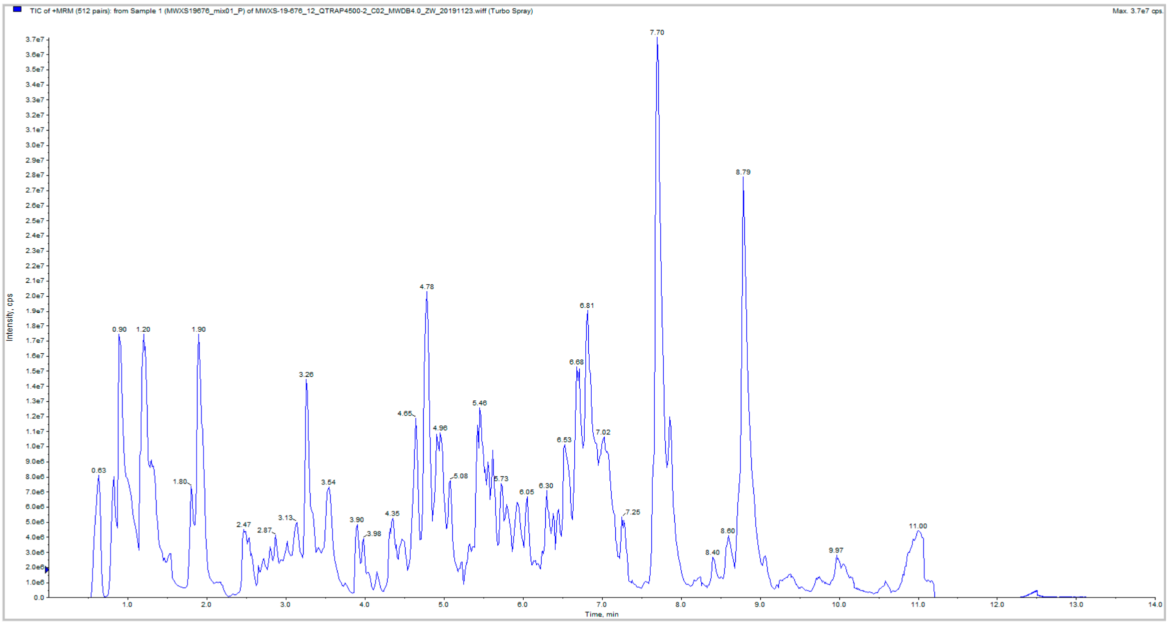

Figure S5 Total ion flow diagram by mixed phase mass spectrometry (**a**: positive ion mode, **b**: negative ion mode). The abscissa is the Retention time (Rt) of metabolite detection, and the ordinate is the ion flow intensity of ion detection (intensity unit: CPS, count per second).

a

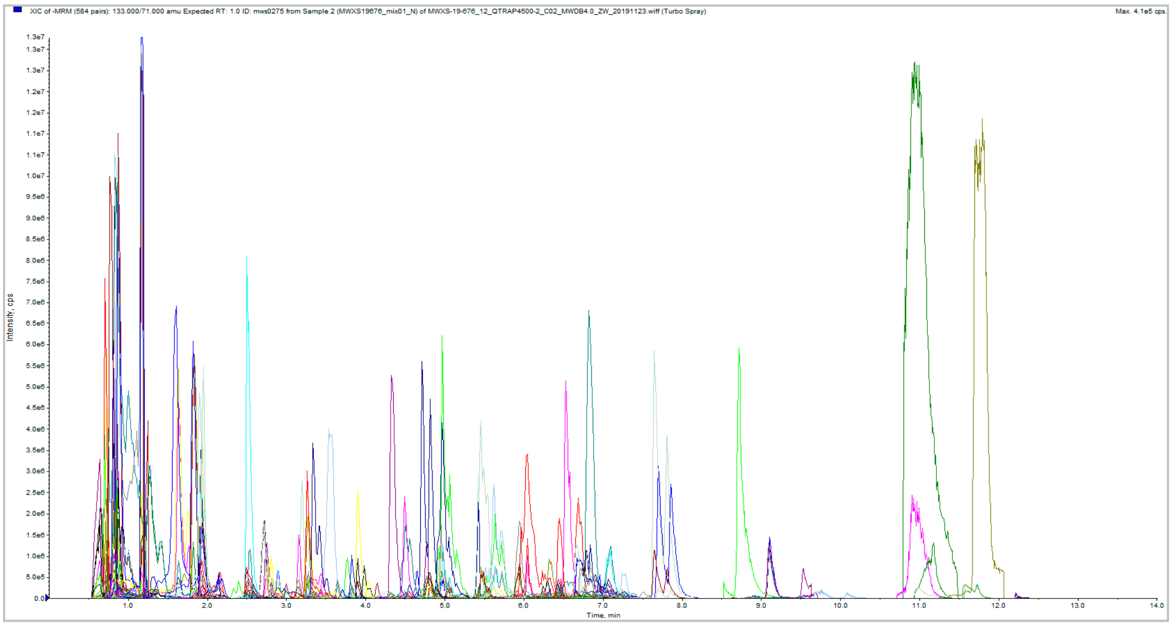

b

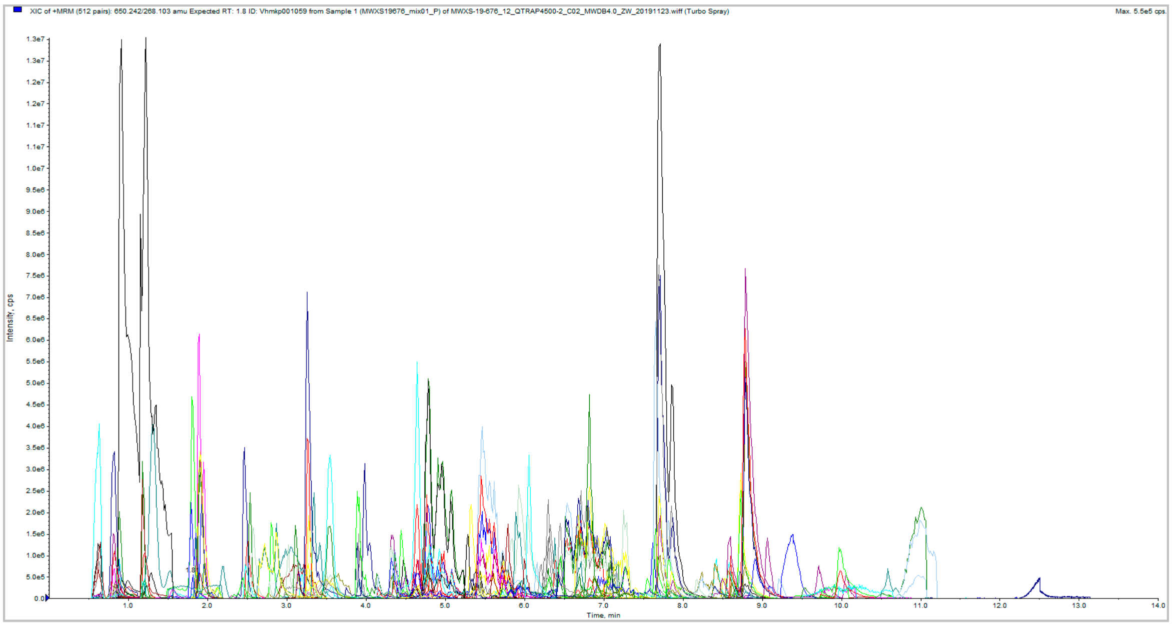

Figure S6 Multi-peak map of MRM metabolite detection (**a**: positive ion mode, **b**: negative ion mode). The abscissa is the Retention time (Rt) of metabolite detection, and the ordinate is the ion flow intensity of ion detection (intensity unit: CPS, count per second).

Table S1. Primers used for qPCR analysis

Abbreviation	Primer sequence (5′-3′)	Description
McOSC1	AACTGGGCTTCGGATCTTGG	*M. charantia McOSC1* primer, forward
McOSC1	CAAACATGGTGCTTGGGCTC	*M. charantia McOSC1* primer, reverse
McOSC2	TGCTGTTCAAGCCATCCTGT	*M. charantia McOSC2* primer, forward
McOSC2	ATGGGCCATCCATGATCTGC	*M. charantia McOSC2* primer, reverse
McOSC3	TGCATCCAGGAAGAATGTGGT	*M. charantia McOSC3* primer, forward
McOSC3	GGAACGTGGGGTGTACAAGT	*M. charantia McOSC3* primer, reverse
McOSC4	TATGATTGGGCCGGAAGCAA	*M. charantia McOSC4* primer, forward
McOSC4	GGCGTGAAGTTCCTGTCTCA	*M. charantia McOSC4* primer, reverse
McOSC5	GGCGTCAAATGGTGGAATGG	*M. charantia McOSC5* primer, forward
McOSC5	TCTTCCTTCCTGTGGCTTGG	*M. charantia McOSC5* primer, reverse
McOSC6	CGATTTGTGGGGCCAATCAC	*M. charantia McOSC6* primer, forward
McOSC6	AAGAGGGGTTCTCCCACGTA	*M. charantia McOSC6* primer, reverse
McOSC7	ACATTACAGGACATCTTGGCACT	*M. charantia McOSC7* primer, forward
McOSC7	ACAAGCATCGGTATCGGGTT	*M. charantia McOSC7* primer, reverse
McOSC8	CTCAACAGGCAGAGAGGGAC	*M. charantia McOSC8* primer, forward
McOSC8	CGATACTCTGCAAGTGCCCA	*M. charantia McOSC8* primer, reverse
McOSC9	CTCTCGCCATGCAAGCTCTA	*M. charantia McOSC9* primer, forward
McOSC9	TGCCATCCATGGTCACAGTC	*M. charantia McOSC9* primer, reverse
β-Tub	CATCTTCCACCTTTACACCCTG	*M. charantia β-Tubulin* primer, forward
β-Tub	CCATGTATCAATCAAACACTCC	*M. charantia β-Tubulin* primer, reverse

Table S2. Summary information of 9 OSC genes in bitter gourd.

Gene name	Accession	Types of OSCs	Protein ID	Gene ID	MW(Da)	PI	GRAVY	NO. of amino acids
McOSC1	XM_022293250.1	CS	XP_022148942.1	LOC111017486	187766.25	4.91	0.739	2289
McOSC2	XM_022282110.1	CAS	XP_022137802.1	LOC111009148	188994.65	4.94	0.657	2298
McOSC3	XM_022279428.1	LAS	XP_022135120.1	LOC111007175	186811.53	4.93	0.749	2280
McOSC4	XM_022299947.1	β-AS	XP_022155639.1	LOC111022720	187912.45	4.91	0.753	2289
McOSC5	XM_022294609.1	β-AS-like	XP_022150301.1	LOC111018501	150619.18	4.98	0.747	1830
McOSC6	XM_022303149.1	LUP-like	XP_022158841.1	LOC111025305	186001.19	4.94	0.736	2265
McOSC7	XM_022303150.1	β-AS-like	XP_022158842.1	LOC111025306	189132.05	4.93	0.743	2292
McOSC8	XM_022294610.1	β-AS-like	XP_022150302.1	LOC111018501	187365.93	4.94	0.734	2268
McOSC9	XM_022301789.1	IMS	XP_022157481.1	LOC111024177	190518.75	4.90	0.803	2301

Notes: MW means relative molecular mass; PI means theoretical isoelectric point; GRAVY means grand average of hydropathicity. The types of OSCs in the table are: β-AS: β-Amyrin synthase; LUP: Lupeol synthase; CAS: Cycloartenol synthase; LAS, lanosterol synthase; CS: Cucurbitadienol synthase; IMS: isomultiflorenol synthase.

Table S3. Ten terpene metabolites detected in transgenic hairy roots

Compounds	Formula	Class	CAS	Fold Change (*GFP* vs. *McOSC7-OE*)
Cucurbitacin D	$C_{30}H_{44}O_7$	Triterpene	3877-86-9	1.43053
Camaldulenic acid	$C_{30}H_{46}O_4$	Triterpene	71850-15-2	2.89626
Enoxolone	$C_{30}H_{46}O_4$	Triterpene	471-53-4	1.82195
Quinovic acid	$C_{30}H_{46}O_5$	Triterpene	465-74-7	4.30638
11-Carbonyl-20β-hydroxycucurbitadienol	$C_{30}H_{48}O_3$	Triterpene	-	0.78697
24,30-Dihydroxy-12(13)-enolupinol	$C_{30}H_{48}O_3$	Triterpene	-	1.16759
2-Hydroxyoleanolic acid	$C_{30}H_{48}O_4$	Triterpene	26707-60-8	1.47516
3,24-Dihydroxy-17,21-semiacetal-12(13) oleanolic fruit	$C_{30}H_{48}O_4$	Triterpene	-	1.47508
Maslinic acid	$C_{30}H_{48}O_4$	Triterpene	4373-41-5	1.45899
Deacetoxycucurbitacin B 3-O-glucoside	$C_{36}H_{54}O_{11}$	Triterpene Saponin	-	1.15847

Notes: Fold Change represents the fold change of the metabolites of transgenic hairy roots compared with the control.
